# Electronic health records to facilitate clinical research

**DOI:** 10.1007/s00392-016-1025-6

**Published:** 2016-08-24

**Authors:** Martin R. Cowie, Juuso I. Blomster, Lesley H. Curtis, Sylvie Duclaux, Ian Ford, Fleur Fritz, Samantha Goldman, Salim Janmohamed, Jörg Kreuzer, Mark Leenay, Alexander Michel, Seleen Ong, Jill P. Pell, Mary Ross Southworth, Wendy Gattis Stough, Martin Thoenes, Faiez Zannad, Andrew Zalewski

**Affiliations:** 1 National Heart and Lung Institute, Imperial College London, Royal Brompton Hospital, Sydney Street, London, SW3 6HP UK; 2 Astra Zeneca R&D, Molndal, Sweden; 3 University of Turku, Turku, Finland; 4 Duke Clinical Research Institute, Durham, NC USA; 5 Servier, Paris, France; 6 Robertson Centre for Biostatistics, University of Glasgow, Glasgow, UK; 7 University of Münster, Münster, Germany; 8 Daiichi-Sankyo, London, UK; 9 GlaxoSmithKline, Stockley Park, UK; 10 Boehringer-Ingelheim, Pharma GmbH & Co KG, Ingelheim, Germany; 11 Optum International, London, UK; 12 Bayer Pharma, Berlin, Germany; 13 Pfizer Ltd., Surrey, UK; 14 Institute of Health and Wellbeing, University of Glasgow, Glasgow, UK; 15 Food and Drug Administration, Silver Spring, MD USA; 16 Campbell University College of Pharmacy and Health Sciences, Campbell, NC USA; 17 Edwards LifeSciences, Nyon, Switzerland; 18 INSERM, Centre d’Investigation Clinique 9501 and Unité 961, Centre Hospitalier Universitaire, Nancy, France; 19 Department of Cardiology, Nancy University, Université de Lorraine, Nancy, France; 20 Glaxo Smith Kline, King of Prussia, Pennsylvania, USA

**Keywords:** Electronic health records, Clinical trials as topic, Pragmatic clinical trials as topic, Cardiovascular diseases

## Abstract

Electronic health records (EHRs) provide opportunities to enhance patient care, embed performance measures in clinical practice, and facilitate clinical research. Concerns have been raised about the increasing recruitment challenges in trials, burdensome and obtrusive data collection, and uncertain generalizability of the results. Leveraging electronic health records to counterbalance these trends is an area of intense interest. The initial applications of electronic health records, as the primary data source is envisioned for observational studies, embedded pragmatic or post-marketing registry-based randomized studies, or comparative effectiveness studies. Advancing this approach to randomized clinical trials, electronic health records may potentially be used to assess study feasibility, to facilitate patient recruitment, and streamline data collection at baseline and follow-up. Ensuring data security and privacy, overcoming the challenges associated with linking diverse systems and maintaining infrastructure for repeat use of high quality data, are some of the challenges associated with using electronic health records in clinical research. Collaboration between academia, industry, regulatory bodies, policy makers, patients, and electronic health record vendors is critical for the greater use of electronic health records in clinical research. This manuscript identifies the key steps required to advance the role of electronic health records in cardiovascular clinical research.

## Introduction

Electronic health records (EHRs) provide opportunities to enhance patient care, to embed performance measures in clinical practice, and to improve the identification and recruitment of eligible patients and healthcare providers in clinical research. On a macroeconomic scale, EHRs (by enabling pragmatic clinical trials) may assist in the assessment of whether new treatments or innovation in healthcare delivery result in improved outcomes or healthcare savings.

Concerns have been raised about the current state of cardiovascular clinical research: the increasing recruitment challenges; burdensome data collection; and uncertain generalizability to clinical practice [1]. These factors add to the increasing costs of clinical research [2] and are thought to contribute to declining investment in the field [1].

The Cardiovascular Round Table (CRT) of the European Society of Cardiology (ESC) convened a two-day workshop among international experts in cardiovascular clinical research and health informatics to explore how EHRs could advance cardiovascular clinical research. This paper summarizes the key insights and discussions from the workshop, acknowledges the barriers to EHR implementation in clinical research, and identifies practical solutions for engaging stakeholders (i.e., academia, industry, regulatory bodies, policy makers, patients, and EHR vendors) in the implementation of EHRs in clinical research.

## Overview of electronic health records

Broadly defined, EHRs represent longitudinal data (in electronic format) that are collected during routine delivery of health care [3]. EHRs generally contain demographic, vital statistics, administrative, claims (medical and pharmacy), clinical, and patient-centered (e.g., originating from health-related quality-of-life instruments, home-monitoring devices, and frailty or caregiver assessments) data. The scope of an EHR varies widely across the world. Systems originating primarily as billing systems were not designed to support clinical work flow. Moving forward, EHR should be designed to optimize diagnosis and clinical care, which will enhance their relevance for clinical research. The EHR may reflect single components of care (e.g., primary care, emergency department, and intensive care unit) or data from an integrated hospital-wide or inter-hospital linked system [4]. EHRs may also change over time, reflecting evolving technology capabilities or external influences (e.g., changes in type of data collected related to coding or reimbursement practices).

EHRs emerged largely as a means to improve healthcare quality [5–7] and to capture billing data. EHRs may potentially be used to assess study feasibility, facilitate patient recruitment, streamline data collection, or conduct entirely EHR-based observational, embedded pragmatic, or post-marketing randomized registry studies, or comparative effectiveness studies. The various applications of EHRs for observational studies, safety surveillance, clinical research, and regulatory purposes are shown in Table 1 [3, 8–10].

**Table 1:** Electronic health records in research

Type	Example	Status
Observational studies	Health utilization Drug utilization Epidemiology (incidence/prevalence) Natural history Risk factors	Widely used and accepted
Safety surveillance	Traditional post-marketing safety surveillance	Widely used and accepted
	Active surveillance (e.g., Sentinel^a^)	Emerging
Clinical research	Hypothesis generation	Accepted
	Feasibility assessments	Accepted
	Performance improvement, guideline adherence	Accepted
	Patient recruitment	Emerging
	Comparative effectiveness, health technology assessments	Emerging
	Pragmatic trials (e.g. PROBE design)	Emerging
	Point of care randomization	Emerging
	Registry randomized trials to test new interventions	Emerging
	Source data to populate eCRF (eliminating or minimizing need for data extraction/data entry)	Emerging/potential
	Endpoint or SAE ascertainment	Emerging/potential
Regulatory	Safety surveillance, pharmacovigilance	Accepted
	New indications or marketing authorization	Potential

^a^Sentinel is the United States Food and Drug Administration’s national electronic system to proactively monitor medical product safety post-marketing, through rapidly and securely accessing data from large amounts of electronic healthcare records, insurance claims, and registries, from a diverse group of data partners [24]

*PROBE* prospective randomized open blinded endpoint, *eCRF* electronic case report form, *SAE* serious adverse event

## Electronic health records for research applications

### Epidemiologic and observational research

EHR data have been used to support observational studies, either as stand-alone data or following linkage to primary research data or other administrative data sets [3, 11–14]. For example, the initial Euro Heart Survey [15] and subsequent Eurobservational Research Program (EORP) [16], the American College of Cardiology National Cardiovascular Data Registry (ACC-NCDR) [14], National Registry of Myocardial Infarction (NRMI), and American Heart Association Get With the Guidelines (AHA GWTG) [17] represent clinical data (collected from health records into an electronic case report form [eCRF] designed for the specific registry) on the management of patients across a spectrum of different cardiovascular diseases. However, modern EHR systems can minimize or eliminate the need for duplicate data collection (i.e., in a separate registry-specific eCRF), are capable of integrating large amounts of medical information accumulated throughout the patient’s life, enabling longitudinal study of diseases using the existing informatics infrastructure [18]. For example, EHR systems increasingly house imaging data which provide more detailed disease characterization than previously available in most observational data sets. In some countries (e.g., Farr Institute in Scotland [19]), the EHR can be linked, at an individual level, to other data sets, including general population health and lifestyle surveys, disease registries, and data collected by other sectors (e.g., education, housing, social care, and criminal justice). EHR data support a wide range of epidemiological research on the natural history of disease, drug utilization, and safety, as well as health services research.

### Safety surveillance and regulatory uses

Active post-marketing safety surveillance and signal detection are important, emerging applications for EHRs, because they can provide realistic rates of events (unlike spontaneous event reports) and information on real-world use of drugs [20]. The EU-ADR project linked 8 databases in four European countries (Denmark, Italy, The Netherlands, United Kingdom) to enable analysis of select target adverse drug events [21]. The European Medicines Agency (EMA) coordinates the European Network of Centres for Pharmacoepidemiology and Pharmacovigilance (ENCePP) which aims to conduct post-marketing risk assessment using various EHR sources [22, 23]. In the United States, the Food and Drug Administration (FDA) uses EHR data from several different sources (e.g., Sentinel and Mini-Sentinel System [24], Centers for Medicare and Medicaid Services [CMS], Veterans Affairs, Department of Defense, Substance Abuse and Mental Health Services Administration) to support post-marketing safety investigations [25].

### Prospective clinical research

National patient registries that contain data extracted from the EHR are an accepted modality to assess guideline adherence and the effectiveness of performance improvement initiatives [26–33]. However, the use of EHRs for prospective clinical research is still limited, despite the fact that data collected for routine medical care overlap considerably with data collected for research. The most straightforward and generally accepted application for EHR is assessing trial feasibility and facilitating patient recruitment, and EHRs are currently used for this purpose in some centers. Using EHR technology to generate lists of patients who might be eligible for research is recognized as an option to meet meaningful use standards for EHR in the United States [6]. However, incomplete data may prohibit screening for the complete list of eligibility criteria [34], but EHRs may facilitate pre-screening of patients by age, gender, and diagnosis, particularly for exclusion of ineligible patients, and reduce the overall screening burden in clinical trials [35]. A second, and more complex, step involves the reuse of information collected in EHRs for routine clinical care as source data for research. Using EHRs as the source for demographic information, co-morbidities, and concomitant medications has several advantages over separately recording these data into an eCRF. Transcription errors may be reduced, since EHR data are entered by providers directly involved in a patient’s care as opposed to secondary eCRF entry by study personnel. The eCRF may be a redundant and costly step in a clinical trial, since local health records (electronic or paper) are used to verify source data entered into the eCRF. Finally, EHRs might enhance patient safety and reduce timelines if real-time EHR systems are used in clinical trials, in contrast to delays encountered with manual data entry into an eCRF. The EHR may facilitate implementation of remote data monitoring, which has the potential to greatly reduce clinical trial costs. The Innovative Medicine Initiative (IMI) Electronic Health Records for Clinical Research (EHR4CR, http://www.ehr4cr.eu) project is one example, where tools and processes are being developed to facilitate reuse of EHR data for clinical research purposes. Systems to assess protocol feasibility and identify eligible patients for recruitment have been implemented, and efforts to link EHRs with clinical research electronic data collection are ongoing [36].

A shift towards pragmatic trials has been proposed as a mechanism to improve clinical trial efficiency [37]. Most of the data in a pragmatic trial are collected in the context of routine clinical care, which reduce trial-specific clinic visits and assessments, and should also reduce costs [38]. This concept is being applied in the National Institutes of Health (NIH) Health Care Systems Research Collaboratory. Trials conducted within the NIH Collaboratory aim to answer questions related to care delivery and the EHR contains relevant data for this purpose. Studies may have additional data collection modules if variables not routinely captured in the EHR are needed for a specific study. Similarly, the Patient-Centered Outcomes Research Institute (PCORI) has launched PCORnet, a research network that uses a common data platform alongside the existing EHR to conduct observational and interventional comparative effectiveness research [9, 39, 40].

The integration of EHRs in the conventional randomized controlled trials intended to support a new indication is more complex. EHRs may be an alternative to eCRFs when data collection is focused and limited to critical variables that are consistently collected in routine clinical care. Regulatory feedback indicates that while a new indication for a marketed drug might be achieved through EHRs, first marketing authorization using data entirely from EHRs would most likely not be possible with current systems until validation studies are performed and reviewed by regulatory agencies. The EHR could also be used to collect serious adverse events (SAE) that result in hospitalization, or to collect endpoints that do not necessarily require blinded adjudication (e.g., death), although the utility of EHRs for this purpose is dependent on the type of endpoint, whether it can reliably be identified in the EHR, and the timeliness of EHR data availability. Events that are coded for reimbursement (e.g., hospitalizations, MI) or new diagnoses, where disease-specific therapy is initiated (e.g., initiation of glucose lowering drugs to define new onset diabetes) tend to be more reliable. The reliability of endpoint collection varies by region and depends on the extent of linkage between different databases.

## Challenges to using electronic health records in clinical trials and steps toward solutions

Challenges to using EHRs in clinical trials have been identified, related to data quality and validation, complete data capture, heterogeneity between systems, and developing a working knowledge across systems (Table 2). Ongoing projects, such as those conducted within the NIH Collaboratory and PCORnet [39, 41] in the United States or the Farr Institute of Health Informatics Research in Scotland, have demonstrated the feasibility of using EHRs for aspects of clinical research, particularly comparative effectiveness. The success of these endeavors is connected to careful planning by a multi-stakeholder group committed to patient privacy, data security, fair governance, robust data infrastructure, and quality science from the outset. The next hurdle is to adapt the accrued knowledge for application to a broader base of clinical trials.

**Table 2:** Challenges of using electronic health records in research

Problem	Example	Potential Solutions
Data quality and validation	Selecting measurement of interest for a clinical trial when multiple measurements are available (e.g., laboratory data) Inaccurate information in EHRs Coding errors	Specific parameters (e.g., using date or time windows) stated in protocol or operating procedures for extracting data from EHR into eCRF Use codes linked to reimbursement, which have greater likelihood of reliability Stakeholder collaboration to develop validation methodology Stakeholder collaboration to contribute data for EHR validation studies
Complete data capture	Clinical endpoints SAEs Problematic in multiple-payer systems Death	Develop standards for data sharing and privacy Explore linking EHRs to national death registries
Heterogeneity among systems	Multiple different vendors within a given country or region Inconfigurable systems Lack of flexible architecture Lack of common data fields, data definitions, and difficulty with data mapping Incomplete data capture Missing fields of interest (i.e. relevant to some diseases but not others) Inability to link systems (i.e. different patient identifiers)	Commit resources to harmonization efforts Form working group with representation from all stakeholders to develop consensus agreement on a common set of data variables to be included in all systems
System knowledge	Inadequate understanding of database and its structure Researchers may not understand limitations of database	Transparency Develop and maintain data standards and operations manuals Report strengths, limitations, and nuances of databases in primary manuscripts Informatics training for investigators

*EHR* electronic health record, *SAE* serious adverse event

### Data quality and validation

Data quality and validation are key factors in determining whether EHRs might be suitable data sources in clinical trials. Concerns about coding inaccuracies or bias introduced by selection of codes driven by billing incentives rather than clinical care may be diminished when healthcare providers enter data directly into the EHRs or when EHRs are used throughout all areas of the health-system, but such systems have not yet been widely implemented [42]. Excessive or busy workloads may also contribute to errors in clinician data entry [43]. Indeed, errors in EHRs have been reported [43–45].

Complete data capture is also a critical aspect of using EHRs for clinical research, particularly if EHRs are used for endpoint ascertainment or SAE collection. Complete data capture can be a major barrier in regions, where patients receive care from different providers or hospitals operating in different EHR systems that are not linked.

Consistent, validated methods for assessing data quality and completeness have not yet been adopted [46], but validation is a critical factor for the regulatory acceptance of EHR data. Proposed validation approaches include using both an eCRF and EHRs in a study in parallel and comparing results using the two data collection methods. This approach will require collaborative efforts to embed EHR substudies in large cardiovascular studies conducted by several sponsors. Assessing selected outcomes of interest from several EHR-based trials to compare different methodologies with an agreed statistical framework will be required to gauge precision of data collection via EHRs. A hybrid approach has also been proposed, where the EHR is used to identify study endpoints (e.g., death, hospitalization, myocardial infarction, and cancer), followed by adjudication and validation of EHR findings using clinical data (e.g., electrocardiogram and laboratory data).

Validity should be defined a priori and should be specific to the endpoints of interest as well as relevant to the country or healthcare system. Validation studies should aim to assess both the consistency between EHR data and standard data collection methods, and also how identified differences influence a study’s results. Proposed uses of EHRs for registration trials and methods for their validation will likely be considered by regulatory agencies on a case-by-case basis, because of the limited experience with EHRs for this purpose at the current time. Collaboration among industry sponsors to share cumulative experiences with EHR validation studies might lead to faster acceptance by regulatory authorities.

The ESC-CRT recommends that initial efforts to integrate EHRs in clinical trials focus on a few efficacy endpoints of interest, preferably objective endpoints (e.g., all-cause or cause-specific mortality) that are less susceptible to bias or subjective interpretation. As noted above, mortality may be incompletely captured in EHRs, particularly if patients die outside of the hospital, or at another institution using a non-integrated EHR. Thus, methods to supplement endpoint ascertainment in the EHR may be necessary if data completeness is uncertain. Standardized endpoint definitions based on the EHR should be included in the study protocol and analysis plan. A narrow set of data elements for auditing should be prospectively defined to ensure the required variables which are contained in the EHR.

Early interaction between sponsors, clinical investigators, and regulators is recommended to enable robust designs for clinical trials aiming to use EHRs for endpoint ascertainment. Plans to translate Good Clinical Practice into an EHR facilitated research environment should be described. Gaps in personnel training and education should be identified and specific actions to address training deficiencies should be communicated to regulators and in place prior to the start of the trial.

### Timely access to electronic health record data

The potential for delays in data access is an important consideration when EHRs are used in clinical trials. EHRs may contain data originally collected as free text that was later coded for the EHR. Thus, coded information may not be available for patient identification/recruitment during the admission. Similarly, coding may occur weeks or months after discharge. In nationally integrated systems, data availability may also be delayed. These delays may be critical depending on the purpose of data extracted from the EHR (e.g., SAE reporting, source data, or endpoints in a time-sensitive study).

### Heterogeneity between systems

Patients may be treated by multiple healthcare providers who operate independently of one another. Such patients may have more than one EHR, and these EHRs may not be linked. This heterogeneity adds to the complexity of using EHRs for clinical trials, since data coordinating centres have to develop processes for interacting or extracting data from any number of different systems. Differences in quality [47], non-standardized terminology, incomplete data capture, issues related to data sharing and data privacy, lack of common data fields, and the inability of systems to be configured to communicate with each other may also be problematic. Achieving agreement on a minimum set of common data fields to enable cross communication between systems would be a major step forward towards enabling EHRs to be used in clinical trials across centers and regions [48, 49].

### Data security and privacy

Privacy issues and information governance are among the most complex aspects of implementing EHRs for clinical research, in part because attitudes and regulations related to data privacy vary markedly around the world. Data security and appropriate use are high priorities, but access should not be restricted to the extent that the data are of limited usefulness. Access to EHR data by regulatory agencies will be necessary for auditing purposes in registration trials. Distributed analyses have the advantage of allowing data to remain with the individual site and under its control [39, 41].

Pre-trial planning is critical to anticipate data security issues and to develop optimal standards and infrastructure. For pivotal registration trials, patients should be informed during the consent process about how their EHRs will be used and by whom. Modified approaches to obtaining informed consent for comparative effectiveness research studies of commonly used clinical practices or interventions may be possible [50]. A general upfront consent stating that EHR data may be used for research is a proactive step that may minimize later barriers to data access, although revision of existing legislation or ethics board rules may be needed to allow this approach. Patients and the public should be recognized as important stakeholders, and they can be advocates for clinical research using EHRs and improve the quality of EHR-based research if they are educated and engaged in the process and the purpose and procedures for EHR use are transparent. Developing optimal procedures for ensuring patients that are informed and protected, balanced with minimizing barriers to research is a major consideration as EHR-based research advances.

### System capabilities

EHRs for use in clinical research need a flexible architecture to accommodate studies of different interventions or disease states. EHR systems may be capable of matching eligibility criteria to relevant data fields and flagging potential trial subjects to investigators. Patient questionnaires and surveys can be linked to EHRs to provide additional context to clinical data. Pre-population of eCRFs has been proposed as a potential role for EHRs, but the proportion of fields in an EHR that can be mapped to an eCRF varies substantially across systems.

EHRs may be more suitable for pragmatic trials where data collection mirrors those variables collected in routine clinical care. Whether regulators would require collection of additional elements to support a new drug or new indication depends on the drug, intended indication, patient population, and potential safety concerns.

### Sustainability

The sustainability of EHRs in clinical research will largely depend on the materialization of their promised efficiencies. Programs like the NIH Collaboratory [41] and PCORnet [39, 41], and randomized registry trials [51, 52] are demonstrating the feasibility of these more efficient approaches to clinical research. The sustainability of using EHRs for pivotal registration clinical trials will depend on regulatory acceptance of the approach and whether the efficiencies support a business case for their use.

## Role of stakeholders

To make the vision of EHRs in clinical trials a reality, stakeholders should collaborate and contribute to the advancement of EHRs for research. Professional bodies, such as the ESC, can play a major role in the training and education of researchers and the public about the potential value of EHR. Clinical trialists and industry must be committed to advancing validation methodology [53]. Investigators should develop, conduct, and promote institutional EHR trials that change clinical practice; such experience may encourage EHR trial adoption by industry and the agencies. Development of core or minimal data sets could streamline the process, reduce redundancy and heterogeneity, and decrease start-up time for future EHR-based clinical trials. These and other stakeholder contributions are outlined in Table 3.

**Table 3:** Role and influence of stakeholders in advancing the use of electronic health records in clinical research

Stakeholder	Contribution
Professional societies	Training and education Global platform for education at annual meetings or congresses Leverage industry support Public education to foster public support Transform EORP into a prospective trial instrument; generate support from industry who may use this resource for future trials Develop data standards (CARDS-revisited) Organize working groups charged with generating common EHR templates or data sets, or achieving agreement on minimum standards Lobby regulatory agencies and industry sponsors
Clinical trialists and industry	Engage other collaborators (e.g., ethicists, CROs, academic CROs, information governance, registries, IT providers, EHR companies, patient advocacy groups, data protection/security experts, legislators/agencies, public funders, legal experts, treating physicians, hospital administrators) Pilot the evaluation of EHR versus conventional non-EHR trials Pilot trials to compare event collection using EHRs versus usual eCRF Conduct actual EHR trials, initially in smaller countries, adapting the approach based on lessons learned, then applying to larger settings Adopt EHRs on an experimental basis for feasibility assessments and patient recruitment Lobby other stakeholders to collaborate towards developing robust methodology to incorporate EHRs in clinical trials Educate professionals and the public about potential value of EHRs
Regulatory	Work with industry to identify appropriate ways to incorporate EHR data prospectively into study designs
EHR vendors	Invest in building research capabilities on EHR platforms

*CARDS* cardiology audit and registration data standards, *CRO* contract research organization, *eCRF* electronic case report form, *IT* information technology, *EHR* electronic health record, *EORP* European Observational Research Program

## Conclusion

Electronic health records are a promising resource to improve the efficiency of clinical trials and to capitalize on novel research approaches. EHRs are useful data sources to support comparative effectiveness research and new trial designs that may answer relevant clinical questions as well as improve efficiency and reduce the cost of cardiovascular clinical research. Initial experience with EHRs has been encouraging, and accruing knowledge will continue to transform the application of EHRs for clinical research. The pace of technology has produced unprecedented analytic capabilities, but these must be pursued with appropriate measures in place to manage security, privacy, and ensure adequacy of informed consent. Ongoing programs have implemented creative solutions for these issues using distributed analyses to allow organizations to retain data control and by engaging patient stakeholders. Whether EHRs can be successfully applied to the conventional drug development in pivotal, registration trials remains to be seen and will depend on demonstration of data quality and validity, as well as realization of expected efficiencies.
